# The Potential of GMP-Compliant Platelet Lysate to Induce a Permissive State for Cardiovascular Transdifferentiation in Human Mediastinal Adipose Tissue-Derived Mesenchymal Stem Cells

**DOI:** 10.1155/2015/162439

**Published:** 2015-10-01

**Authors:** Camilla Siciliano, Isotta Chimenti, Antonella Bordin, Donatella Ponti, Paola Iudicone, Mariangela Peruzzi, Erino Angelo Rendina, Antonella Calogero, Luca Pierelli, Mohsen Ibrahim, Elena De Falco

**Affiliations:** ^1^Department of Medical-Surgical Sciences and Biotechnologies, Faculty of Pharmacy and Medicine, “Sapienza” University of Rome, Corso della Repubblica 79, 04100 Latina, Italy; ^2^Center for Life Nano Science@Sapienza, Istituto Italiano di Tecnologia, Viale Regina Elena 291, 000161 Rome, Italy; ^3^Immunohematology and Transfusion Medicine, San Camillo Forlanini Hospital, Circonvallazione Gianicolense 87, 00152 Rome, Italy; ^4^Department of Medical-Surgical Science and Translational Medicine, Division of Thoracic Surgery, “Sapienza” University of Rome, Sant'Andrea Hospital, Via di Grottarossa 1035, 00189 Rome, Italy; ^5^Department of Experimental Medicine, “Sapienza” University of Rome, Viale Regina Elena 324, 000161 Rome, Italy

## Abstract

Human adipose tissue-derived mesenchymal stem cells (ADMSCs) are considered eligible candidates for cardiovascular stem cell therapy applications due to their cardiac transdifferentiation potential and immunotolerance. Over the years, the in vitro culture of ADMSCs by platelet lysate (PL), a hemoderivate containing numerous growth factors and cytokines derived from platelet pools, has allowed achieving a safe and reproducible methodology to obtain high cell yield prior to clinical administration. Nevertheless, the biological properties of PL are still to be fully elucidated. In this brief report we show the potential ability of PL to induce a permissive state of cardiac-like transdifferentiation and to cause epigenetic modifications. RTPCR results indicate an upregulation of Cx43, SMA, c-kit, and Thy-1 confirmed by immunofluorescence staining, compared to standard cultures with foetal bovine serum. Moreover, PL-cultured ADMSCs exhibit a remarkable increase of both acetylated histones 3 and 4, with a patient-dependent time trend, and methylation at lysine 9 on histone 3 preceding the acetylation. Expression levels of p300 and SIRT-1, two major regulators of histone 3, are also upregulated after treatment with PL. In conclusion, PL could unravel novel biological properties beyond its routine employment in noncardiac applications, providing new insights into the plasticity of human ADMSCs.

## 1. Introduction

Advances in stem cell therapy for treating cardiovascular diseases have been hampered by the complexity to understand the modality by which stem cells are committed to the cardiovascular lineage once injected and what molecular mechanisms underlie stem cell regenerative potential. In combination with the transdifferentiation ability toward the cardiac lineage, stem cell candidates should have also additional attributes such as high achievable yield, reproducible in vitro expansion, and immunotolerance. Among those adult stem populations under clinical investigation, mesenchymal stem cells (MSCs) have been reported to show all the above-mentioned features [1–4]. Particularly, adipose tissue-derived MSCs (ADMSCs), which originate from a noncardiac tissue source, have been successfully employed as a tool for cardiac stem cell-based therapy and for myocardial infarct treatment [5–7].

ADMSCs are routinely expanded in platelet lysate (PL), a hemocomponent enriched with growth factors and cytokines already employed as a substitute for foetal bovine serum (FBS) in cell culture and for treating those medical applications where repair, regeneration, and neoangiogenesis are desirable [8–10]. Interestingly, few studies have recently suggested PL as a candidate for cardiovascular tissue engineering purposes, due to its ability to enhance both matrix production and remodelling [11]. In addition, 5-azacitidine and PL-cultured ADMSCs have been demonstrated to possess high ability to transdifferentiate towards the cardiac phenotype similarly to FBS [12] and to promote migration and differentiation of murine cardiac fibroblasts [13]. Therefore, PL could represent an interesting tool for cardiac repair potential of ADMSC cultures.

We have already described both the suitability of the GMP-compliant PL to enhance the biological properties of a novel tissue source of human ADMSCs derived from the mediastinal depots [8] and their ability to transdifferentiate towards a cardiac-like phenotype upon exposure to a niche-like microenvironment such as that produced by cardiospheres [14], a 3D in vitro model of spontaneous cardiac microtissue [15–19].

In this brief report, we show for the first time the ability of a GMP-compliant PL (Mesengen kindly provided by Futura Relife, Publication number WO/2013/042095) to potentially promote a permissive state of cardiovascular commitment in human mediastinal ADMSCs and to cause epigenetic modifications mainly based on acetylation of histones 3 and 4.

## 2. Materials and Methods

### 2.1. Isolation and Expansion of Human Mediastinal ADMSCs

Human mediastinal ADMSCs have been isolated from patients undergoing thoracic surgery, as previously described [8, 14]. Informed consent was obtained from all subjects before the procedure. Experiments have been conducted in compliance with the Tenets of the Declaration of Helsinki. Briefly, after enzymatic digestion with trypsin-EDTA/1 mg/mL collagenase (Aurogene, Rome, Italy, Cat. number AU-X0930-100; GIBCO, Monza, Italy, Cat. number 17100), cells were divided into equal amounts and seeded in complete medium (DMEM-low glucose/1% PenStrep/1% glutamine/1% nonessential amino acids, all from Biowest, Nuaillé, France) supplemented either with 10% GMP-compliant PL (Mesengen kindly provided by Futura Relife S.r.l. Publication number WO/2013/042095) or FBS (Sigma-Aldrich, St. Louis, MO, USA), at a density of 4000 cells/cm^2^. Nonadherent cells were removed after 3 days. Cell count and viability were evaluated by Trypan Blue (Sigma-Aldrich, St. Louis, MO, USA, Cat. number T8154). ADMSC cultures were characterized according to the criteria of the International Society for Cellular Therapy (ISCT) [20]. All experiments were performed at passage 3.

### 2.2. GMP-Compliant Platelet Lysate Preparation

GMP-compliant PL (Mesengen) was prepared and virally inactivated, as previously described [8, 9]. Briefly, after obtaining informed consent, buffy coats from healthy volunteers were centrifuged and treated with InterSol solution (318 mg Na-citrate 2H_2_O, 305 mg Na 2-phosphate anhydro, 105 mg Na dihydrogen phosphate 2H_2_O, 442 mg Na-acetate 3H_2_O, 452 mg NaCl, 100 mL H_2_O, Fenwal Inc., Lake Zurich, Illinois) and subsequently with 20–30% human plasma. Potential pathogens were inactivated by using the Intercept Blood System for Platelets (Cerus Corporation, Concord, California, USA). PL was stored at −80°C for 24 hours before thawing at 37°C for 60 minutes. This procedure was repeated three times to enrich the pool of growth factors. Concentration and sterility of the preparation were determined by the haematology analyzer ABX Pentra DX 120 (Horiba ABX, Montpellier, France) and BACTEC 9240 (Becton and Dickinson), respectively. 5 U/mL heparin was added to cell culture media in order to avoid fibrin gel formation.

### 2.3. Real Time PCR

Total RNA was extracted using Total RNA Extraction kit (Qiagen) according to manufacturer's instructions and then reverse-transcribed using High Capacity cDNA Reverse Transcription kit (Applied Biosystems). The evaluation of the expression of cardiac transdifferentiation genes [14] was assessed by quantitative Real Time PCR [14] using SensiMix SYBR Hi-ROX kit (Bioline) on a 7900HT Fast Real Time PCR System equipped with SDS software (Applied Biosystems) for 40 thermal cycles (95°C for 15 s, 56/58°C for 15 s, and 72°C for 15 s). Primers sequence and annealing temperatures for cardiac markers have been used according to our previous publication [14], whereas p300 and SIRT-1 primers sequences were the following: p300 forward → GGTCAAGCTCCAGTGTCTCAA; reverse → CCCTGGAGGCATTATAGGAGA; SIRT-1 forward → TGTACGACGAAGACGACGAC; reverse → TTCATCACCGAACAGAAGGTT.

The ΔΔCt method was used for data analysis, with GAPDH as the housekeeping gene and FBS as the reference condition.

### 2.4. Immunofluorescence

Human mediastinal ADMSCs cultured in PL or FBS were fixed with 4% paraformaldehyde (Sigma-Aldrich, St. Louis, MO, USA, Cat. number 158127). After permeabilization with 0.5% Triton X-100 (Sigma) and incubation in blocking buffer (0.2% Gelatin, Sigma), primary antibodies (SMA 1 : 100 Abcam Cat. number ab7817; c-kit 25 *μ*g/mL Abcam Cat. number ab5506; Thy-1 1 : 50 Dianova Cat. number DIA 120; Cx43 1 : 50 Millipore Cat. number MAB3067) were added overnight at +4°C [14]. Secondary antibodies (all Alexa Fluor 488, Invitrogen) were added at room temperature for 2 hours. Images were acquired by fluorescent microscope (Leica, software IAS2000). Nuclei were counterstained by DAPI (1 : 1000, 4′-6′-diamidino-2-phenylindole, powder ≥ 98%; Sigma, St. Louis, MO, USA, Cat. number D9542).

### 2.5. Acetylated Histones 3 and 4 Quantification

The endogenous levels of acetylated histones 3 and 4 were determined by ELISA assay (PathSCan acetyl-histone 3 and histone 4 Sandwich ELISA kit, Cell Signaling Cat. numbers 7209 and 7238, resp.) according to manufacturer's instructions. Briefly, human ADMSCs after 0, 6, 12, 24, 48, and 72 hours of stimulation with 10% LP were lysed with acid buffer and protein quantified by Bradford method. Equal amounts of protein lysates were incubated on histones 3 and 4 coated microwells. After washing, the acetylated-lysine monoclonal antibody was added, followed by incubation with the peroxidase-linked (HRP) antibody. The substrate for the HRP antibody was then added to develop colour. The absorbance was measured at 540 nm on a 96-well plate reader (Tecan). The magnitude of the optical density is directly proportional to the quantity of acetylated histones 3 and 4. Stimulation with Valproic acid (15 mM for 12 hours) was used as positive control.

### 2.6. Immunoblotting

Protein expression was analysed by denaturing discontinuous gel electrophoresis (Laemmli Gel Method). Whole extracts were obtained from subconfluent cultures resuspending cells in RIPA buffer (25 mM Tis-HCl pH 7.6, 150 mM NaCl, 1% NP-40, 0,1% SDS, 1% deoxycholate, protease inhibitor (Sigma), and 0,1% mM phenylmethylsulfonyl fluoride). After incubation for 30 min at 4°C the lysate was centrifuged at 9240 rcf for 10 min. Protein concentration was determined by using Bradford reagent (Biorad). Whole extracts (80 *μ*g) were fractionated by 15% SDS-PAGE electrophoresis (acrylamide and bisacrylamide 29 : 1, Biorad) and transferred onto 0.45 m PVDF sheets (Amersham) by using Transfer Blot SD Cell (Biorad) at 15 Volts for 30 min in presence of Towbin buffer (25 mM Tis-HCl, 192 mM glycine pH 8.3, 20% methanol, and 0.1% SDS). Membrane was blocked in 5% nonfat dry milk (Cell Signaling) and incubated overnight at 4°C with primary antibody against bimethylation sites of lysine 9 on histone 3, anti-H3K9m2 (07-212 Millipore), diluted 1 : 1000 in 5% nonfat dry milk. After incubation the sheets were washed in PBST buffer and incubated at room temperature for 1 hour in secondary antibody conjugated to horseradish-peroxidase (Amersham) diluted 1 : 10000. Actin was used as loading control. After ECL assay (GE Healthcare) the membrane was incubated with film specific for protein detection (Kodak).

### 2.7. Statistical Analysis

Data are presented as mean ± SEM unless specified. The independent sample two-tailed *t*-test with associated 95% confidence intervals was used to compare any two groups. A *p* value < 0.05 has been considered statistically significant.

## 3. Results and Discussion

**Figure 1:**
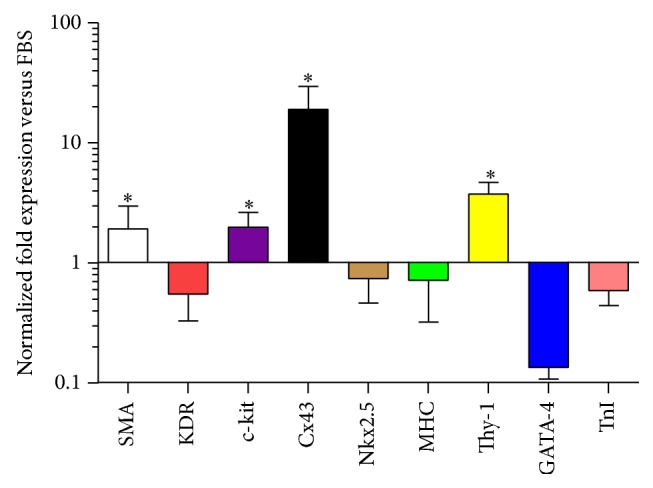
Cardiovascular gene expression profile by RTPCR of ADMSCs stimulated with 10% GMP-compliant platelet lysate. The graph shows a significant upregulation of SMA, c-kit, Cx43, and Thy-1. ADMSCs cultured in FBS were considered as the reference for normalization. ^*∗*^ *p* < 0.05.

In order to investigate whether the GMP-compliant PL is able to induce cardiac-like transdifferentiation, we derived three human primary mediastinal ADMSC lines from bioptic samples, expanded in presence of 10% PL (which is the standard concentration employed in vitro) or in presence of standard FBS and differentially characterized as we previously described [8, 14]. At passage 3, we evaluated their cardiac gene expression profile induced by PL, by testing cardiac stem cell/differentiation genes, such as smooth muscle actin (SMA), KDR, c-kit, Connexin 43 (Cx43), Nkx2.5, myosin heavy chain (MHC), Thy-1, GATA-4, and troponin I (TnI). ADMSCs cultures derived from biopsies cultured in FBS were used as control. Results showed a significant upregulation of SMA, c-kit, Cx43, and Thy-1 in PL cultures versus FBS (Figure 1, all *p* < 0.05). Differently, other cardiac markers (KDR, Nkx2.5, MHC, GATA-4, and TnI) were unmodified.

**Figure 2:**
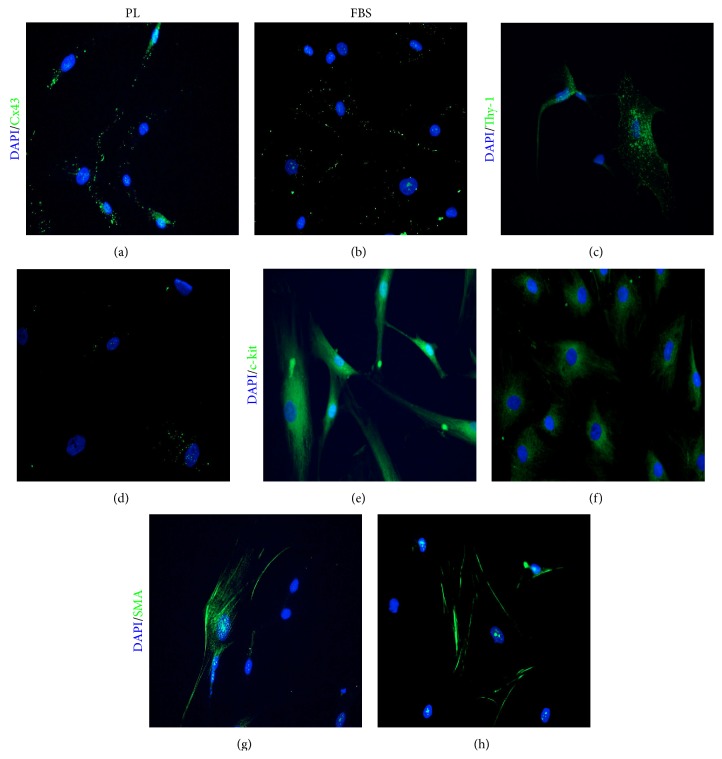
Representative images of immunofluorescence staining for SMA, Cx43, Thy-1, and c-kit. Platelet lysate-cultured ADMSCs displayed positive aligned and focal-like fluorescence for Cx43 (a) and positivity for Thy-1 (c) and c-kit (e). ADMSCs cultured in the presence of FBS showed a scattered distribution of Cx43 (b) and a fainter positivity for Thy-1 (d) and c-kit (f). The expression of SMA was similar in both conditions (g-h). DAPI staining (blue); Cx43, Thy-1, c-kit, and SMA staining: green. PL: platelet lysate. Magnification 20x.

To then verify whether a similar phenotype was also expressed at protein level, we performed immunofluorescence staining for the upregulated cardiovascular markers (SMA, Cx43, Thy-1, and c-kit). Results showed a much more aligned distribution of Cx43, with focal-like areas in PL-cultured ADMSCs, compared to scattered spots in FBS (Figure 2(b)). We also found an enhanced expression of Thy-1 and c-kit (Figures 2(c)–2(e)) compared to FBS (Figures 2(d)–2(f)) but similar positivity for SMA (Figures 2(g) and 2(h)).

The differentiation of ADMSCs toward a cardiogenic phenotype has been described, spontaneously [21], by using 5-azacytidine [22] or by culturing MSCs onto a feeder layer of rat cardiomyocytes [23]. Recently, we have strengthened the biological potential of a novel multipotent stem cell population of ADMSCs derived from the mediastinal depots by demonstrating their plastic commitment to cardiac/endothelial/muscular-like phenotypes upon cardiosphere-mediated paracrine action [14]. Our results confirm such plasticity of human ADMSCs, able to respond to a wide range of stimuli including PL. Nevertheless, cardiosphere and PL-generated microenvironments differently influenced ADMSC cultures. Specifically, both c-kit and Cx43 (a stem cell cardiac precursor marker, albeit highly debated [24], and a late marker of cardiac transdifferentiation, resp.) are upregulated in PL-cultured ADMSCs, but they were not induced by cardiosphere-conditioned media exposure. This indicates the relevance of the specific stimulus on the outcome of the phenotypical commitment, most likely due to the different composition in the type and concentration of soluble factors within the two microenvironments [25–27]. Besides, although a different study has already suggested the potential of PL to promote cardiac phenotype of ADMSCs [12], the commitment was driven by a combination of PL and 5-azacytidine (a widely used, although controversial, cardiac transdifferentiation agent [14]), thus masking the real efficacy of PL. The GMP-compliant PL preparations employed in this study are enriched with specific growth factors, including VEGF, TGF-*β*1, PDGF, and EGF [8, 9], known to improve cell proliferation and migration, the angiogenic process, and collagen deposition and recruitment of circulating macrophages and lymphocytes to the site of injury, thus enhancing tissue regeneration and wound healing [23, 28–31]. In particular, TGF-*β*1 has been reported to significantly increase the expression of both Cx43 and SMA [32] and to contribute to the remodeling of cardiac fibroblast populations after myocardial infarct [33]. The upregulation of Cx43 and SMA expression levels in ADMSCs upon PL stimulation confirms the role of PL in the cytoskeletal rearrangement and suggests an electrical and metabolic coupling potential between MSCs. To what extent PL could lead to beneficial effects is still to be fully elucidated. For instance, the culture of heart valve tissue constructs in presence of PL is not desirable due to fibrotic effects [34], thus suggesting a selective suitability for cardiovascular applications.

Differently, despite a discrete concentration of VEGF and EGF in the PL preparations used in this study, we have not observed a preferential commitment to the endothelial lineage but to an increase in c-kit gene expression levels, which is normally not expressed by ADMSCs after isolation [8]. However, ADMSCs have been shown to contain a c-kit^+^ subpopulation, probably derived by the perivascular area and displaying high preservation ability for cardiac progenitor cells [35]. Besides, considering that niches containing a combination of adult cardiomyocytes, allogenic MSCs, and c-kit^+^ cardiac progenitor cells expressing Cx43-mediated gap junctions have been found in in vivo models [36, 37], we could speculate that the treatment with PL might also promote a protective and feeder-like support role for ADMSCs within the cardiac niche once injected.

**Figure 3:**
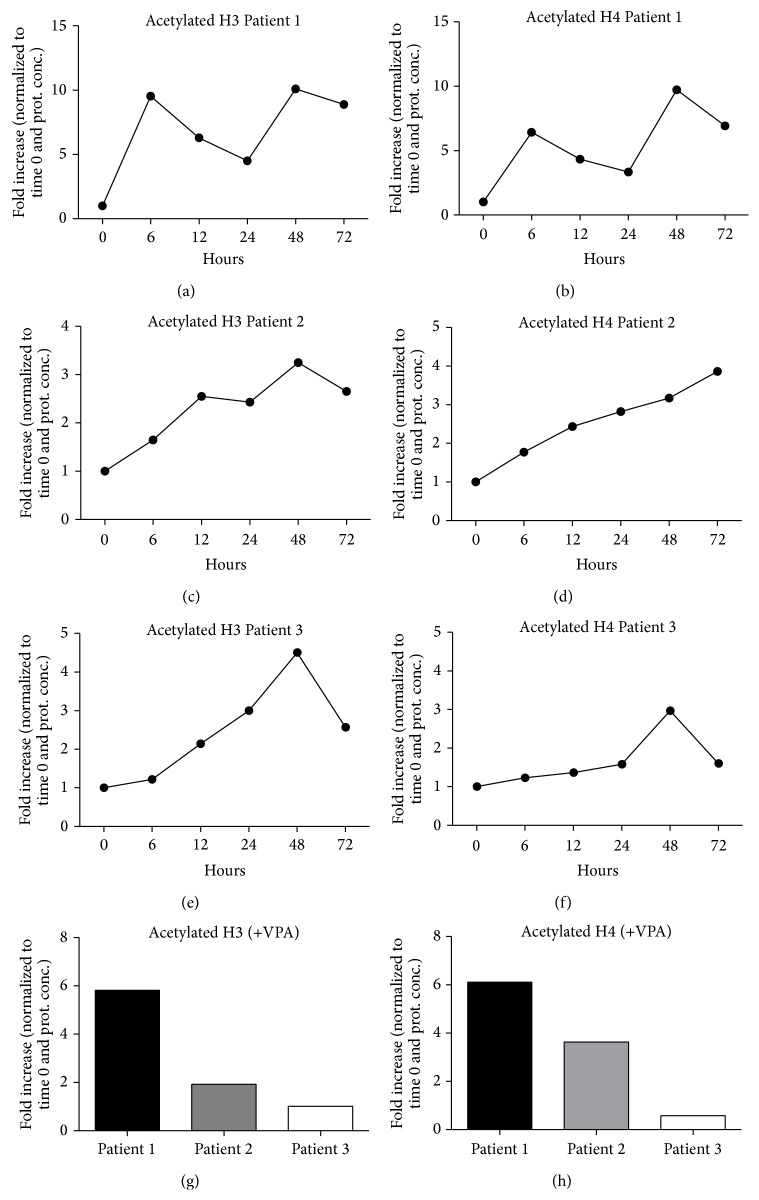
Detection of acetylated histone 3 and histone 4 levels in Patients 1 (a, b), 2 (c-d), and 3 (e-f) up to 72 hours of stimulation with platelet lysate, by Sandwich ELISA Antibody Pair assay. The graphs show an oscillatory response to platelet lysate of Patient 1 and a sustained signal in Patients 2 and 3 for both histones. Levels of acetylated H3 and H4 in Patients 1, 2, and 3 after stimulation with Valproic acid (VPA) showed a response with a different reactivity of the subjects (g-h). Results have been normalized to both total protein concentration and time 0. H3, histone 3; H4, histone 4.

Finally, given the involvement of histones 3 and 4 modifications in cardiac differentiation [38], we have assessed whether PL could induce similar epigenetic changes. Accordingly, human mediastinal ADMSC cultures derived from 3 patients were starved for 2 hours and stimulated with 10% PL up to 72 hours and both levels of total acetylated histones 3 and 4 (occurring at lysine residues such as 4, 9, 14, 18, 23, or 27) [39, 40] were screened by ELISA assay. Conditioning with Valproic acid, known to exert histone deacetylase-inhibiting effects, was used as positive control. Results showed a significant increase in histone 3 acetylation in all patients, but with a different time trend, certainly due to their individual genetic asset which influences the biological response to PL. Patient 1 displayed an oscillating trend starting with a prompt upregulation of histone 3 acetylation after 6 hours (Figure 3(a)), whereas Patients 2 and 3 responded with a more constant increase over the time, reaching a peak after 48 hours (Figures 3(c) and 3(e)). A similar trend with regard to acetylated histone 4 was observed in all patients (Figures 3(b), 3(d), and 3(f)). The interpatient variability was also confirmed by the different reactivity to Valproic acid (Figures 3(g) and 3(h)).

**Figure 4:**
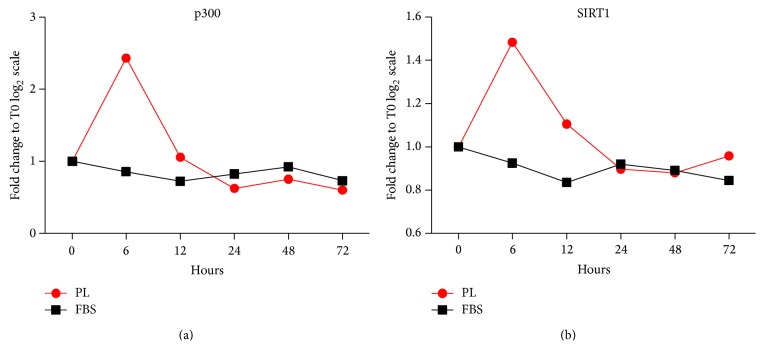
Evaluation of p300 and SIRT-1 gene expression by RTPCR of ADMSCs derived from Patient 3 and stimulated with 10% GMP-compliant platelet lysate. The graphs show that the treatment with platelet lysate but not with FBS is able to increase mRNA expression levels of both p300 (a) and SIRT-1 (b) after 6 hours, rapidly decreasing and fluctuating around the baseline. PL, platelet lysate.

Next, we investigated whether p300 and SIRT-1, two main regulators of histone 3 with acetyltransferase [41] and deacetylase activity [42], respectively, were modulated upon PL treatment. To this aim, we specifically focused on Patient 3, who had displayed the lowest reactivity to Valproic acid, thus potentially suggesting a more complex chromatin structure. Results showed that the treatment with PL, but not with FBS, led to increased mRNA expression levels of both p300 and SIRT-1 after 6 hours, rapidly decreasing and fluctuating around the baseline (Figures 4(a) and 4(b)). The discrepancy between the early upregulation of p300 and the late acetylation of histone 3 (Figure 3(e)) could be explained by a possible autoacetylation of the protein, as already observed in cardiac myocytes upon acute stress, leading to the stabilization and accumulation of p300 itself after 24 hours [43]. Moreover, a parallel involvement of SIRT-1 is not surprising, considering that other cardiac-like genes such as MHC, KDR, TnI, and GATA-4 were downregulated (Figure 1), thus suggesting control of epigenetic gene silencing through deacetylase activity. Although the mechanism by which SIRT-1 acts in MSCs is still unclear, the loss of SIRT-1-based deacetylase activity corresponds to deregulated MSC differentiation [44, 45]. Therefore, MSC lineage determination is certainly controlled by both epigenetic histone acetylation and deacetylation mechanisms.

**Figure 5:**
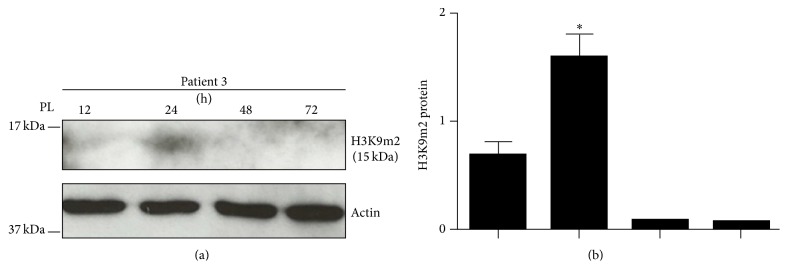
Representative image of the immunoblot of the bimethylation on lysine 9 of H3 (H3K9m2) in Patient 3 (a), where a significant increase at 24 hours was observed (b). ^*∗*^ *p* < 0.05; PL, platelet lysate; H3, histone 3.

Finally, given the main role of acetylated histone 3 to mediate cardiac differentiation, we asked whether a modulation of the methylation, also known to control mesodermal transdifferentiation in MSCs [46], could occur after stimulation with PL. To this aim, we specifically assessed the bimethylation of lysine 9 on histone 3 (H3K9m2), normally associated with the chondrogenic, osteogenic, and adipogenic transdifferentiation ability of MSCs [46]. Results on Patient 3 have shown a significant increase in methylation at 24 hours after stimulation with PL (Figures 5(a) and 5(b)).

The physiological process of transdifferentiation occurs at gene expression level, influencing stem cell reprogramming and lineage commitment [47, 48]. Particularly, epigenetic modifications by regulation of histone acetylation and methylation have been associated with stem cell fate, determining the modality by which chromatin is more or less accessible at the gene promoters sites [49]. A recent study has demonstrated that distinct gene-specific histone modifications are involved in cardiac differentiation potential of cardiac progenitor cells (CPCs) and that histones 3 and 4 of CPCs^Sca-1+/CD29+^ are more acetylated at the promoters of cardiac specific genes, thus highlighting that both these two histone proteins are involved in cardiac differentiation [38]. Interestingly, our data highlight that PL is able to induce epigenetic modifications by promoting more transcriptionally active chromatin structures and that the response is patient-dependent.

A further study has reported that the epigenetic cardiac signature in ADSCMs is based on a combination of low levels of total acetylated histone 3 and high levels of trimethylated lysine 27 [50], whereas a different study has associated the same methylation (H3K27) with the osteogenic differentiation and the acetylation of histones 3 and 4 mainly with the cardiac lineage [46]. This clear discrepancy is likely due to the possibility that MSCs derived from different tissue sources do not share similar epigenetic states [22], thus suggesting a consequent different transdifferentiation potential. By conditioning with PL, we also observed a general acetylation of histone 3 peaking at 48 hours preceded by dimethylation of lysine 9. We might speculate that the shift towards the demethylation, which is associated with a condensed state of histone 3 [46], could represent a preliminary and permissive step to switch on the acetylation. In addition, considering the bivalent activity of the chromatin in MSCs where parallel methylation and acetylation can coexist [50] but also the role of the demethylation of lysine 9 on histone 3 both in adipogenic, chondrogenic, and osteogenic transdifferentiation [46, 51, 52] and in the capacity to escape senescence mechanisms [53], we could suggest that PL is able to target a multipotent gene expression potential in ADMSCs, including the cardiac lineage but concurrently preserving a proliferative stem cell pool and the capacity to transdifferentiate into the mesodermal lineage. In other words, the commitment to the cardiovascular and the mesodermal lineage would not be mutually exclusive. Moreover, our data are in line with a further study, showing that the inhibition of histone acetylation interferes with the mesodermal commitment of PL-grown ADMSCs [54], thus confirming that the acetylation of histone 3 can bear a bivalent function in inducing both cardiac and mesodermal commitment. Since stem cell fate is not solely determined by epigenetic modifications, the functional consequence of the potential ability of PL to induce either cardiovascular or mesodermal transdifferentiation will eventually depend on the tissue microenvironment surrounding the ADMSCs.

We cannot still conclude that the potential of PL is sufficient to the final cardiac transdifferentiation in ADMSCs but only that it is able to provide a permissive state, likely functional upon specific cardiac/vascular signals. Besides, we could assume that the chromatin remodelling observed is due to a general effect caused by the synergic action of the numerous growth factors within the PL or simply that other nonepigenetic mechanisms, such as posttranslational histone modifications, are involved. Moreover, a proper comparison with a nonadipose tissue-derived MSC population and with FBS would allow us to understand whether or not the effect is specific to both ADMSC and PL.

The possibility to precondition MSCs prior to in vivo injection with PL (containing numerous angiogenic and muscular growth factors but many inflammatory cytokines as well) could represent an advantage compared to other differentiation methods in modulating the immune response. Accordingly, in order to enhance MSC cardiac commitment and therapeutic potential, PL could substitute the numerous current attempts to design cocktails of soluble molecules and factors [55, 56] by a more standardized, safe, and reproducible method. The numerous intrinsic biological effects of MSCs could be maximized and potentiated, including the feeder-like support potential of MSCs on cardiac myocytes, considering the ability of MSCs to secrete TGF-*β* and its discrete amount in PL preparations that may allow cardiac myocytes to reenter the cell cycle [55]. The dual ability of PL to both simultaneously expand and commit ADMSCs to the cardiac-like lineage could be also useful to overcome the low retention of engrafted MSCs observed within the cardiac tissue after in vivo injection [57], compensated by a higher number of injected cells. Besides, it has been extensively demonstrated that the cardiac differentiation potential of other different stem cell types (bone marrow derived c-kit^+^ and monocytes, fibroblasts, or cardiomyocytes) is limited as they only provide either the angiogenic or the muscular component [58]. From this perspective, only cardiac resident progenitor cells could represent a better suitable option than MSCs [14, 59, 60]; however their scarcity and difficult retrievability in cardiac tissue limit their employment, albeit even combined cell therapy protocols have been suggested [61]. Considering the intrinsic ability of ADMSCs to differentiate into multiple cell types including muscle cells [62] and to induce neovascularization in the injured heart [63], the treatment with PL may be biologically significant on multiple levels in MSCs.

## 4. Conclusions

Understanding the mechanisms controlling the cardiac transdifferentiation process in ADMSCs is essential to boost MSC unique beneficial properties. The possibility to explore PL as a cardiac transdifferentiation permissive agent could suggest a novel therapeutic approach beyond its routine employment in noncardiac applications and provide new insights into the biology of human mediastinal ADMSCs.

## Supplementary Material

After fixing with 4% paraformaldehyde (Sigma-Aldrich, St. Louis, MO, USA Cat. N. 158127). ADMSC cultures were permeabilized with 0.5% Triton X-100 (Sigma) and incubated in blocking buffer (0.2% Gelatin, Sigma) as described for primary antibodies [14]. Negative controls consisted of the secondary antibody alone (all AlexaFluor 488, Invitrogen) added at room temperature for 2 hours. Images were acquired by fluorescent microscope (Leica, software IAS2000). Nuclei were counterstained by DAPI (1:1000, 4'-6'-Diamidino-2-phenylindole, powder ≥98%; Sigma, St. Louis, MO, USA, Cat. N. D9542).
