# Supplementary figures and images for: The Potential of GMP-Compliant Platelet Lysate to Induce a Permissive State for Cardiovascular Transdifferentiation in Human Mediastinal Adipose Tissue-Derived Mesenchymal Stem Cells
Source: Biomed Res Int. 2015 Oct 1;2015:162439. doi: 10.1155/2015/162439 (PMC4606096; doi:10.1155/2015/162439)

DAPI//Ab

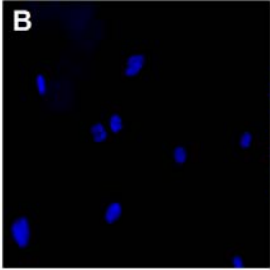

DAPI//Ab

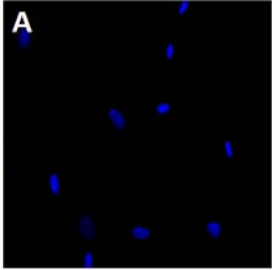

Supplement: Supplementary file 1 — After fixing with 4% paraformaldehyde (Sigma-Aldrich, St. Louis, MO, USA Cat. N. 158127). ADMSC cultures were permeabilized with 0.5% Triton X-100 (Sigma) and incubated in blocking buffer (0.2% Gelatin, Sigma) as described for primary antibodies [14]. Negative controls consisted of the secondary antibody alone (all AlexaFluor 488, Invitrogen) added at room temperature for 2 hours. Images were acquired by fluorescent microscope (Leica, software IAS2000). Nuclei were counterstained by DAPI (1:1000, 4'-6'-Diamidino-2-phenylindole, powder ≥98%; Sigma, St. Louis, MO, USA, Cat. N. D9542). [file 162439.f1.pdf]
